# Pharmacokinetic-Pharmacodynamic Model of Newly Developed Dexibuprofen Sustained Release Formulations

**DOI:** 10.5402/2012/451481

**Published:** 2012-12-06

**Authors:** Selvadurai Muralidharan

**Affiliations:** Faculty of Pharmacy, AIMST University, Semeling, 08100 Bedong, Malaysia

## Abstract

Pharmacokinetic-pharmacodynamic (PK-PD) modeling has emerged as a major tool in clinical pharmacology to optimize drug use by designing rational dosage forms and dosage regimes. Quantitative representation of the dose-concentration-response relationship should provide information for the prediction of the level of response to a certain level of drug dose. This paper describes the experimental details of the preformulation study, tablet manufacture, optimization, and bioanalytical methods for the estimation of dexibuprofen in human plasma. The hydrophilic matrix was prepared with xanthen gum with additives Avicel PH 102. The effect of the concentration of the polymer and different filler, on the *in vitro* drug release, was studied. Various pharmacokinetic parameters including AUC_0–*t*_, AUC_0–∞_, *C*_max_, *T*_max_, *T*_1/2_, and elimination rate constant (*K*_el_) were determined from the plasma concentration of both formulations of test (dexibuprofen 300 mg) and reference (dexibuprofen 300 mg tablets). The merits of PK-PD in the development of dosage forms and how PK-PD model development necessitates the development of new drugs and bio analytical method development and validation are discussed. The objectives of the present study, namely, to develop and validate the methods to estimate the selected drugs in the biological fluids by HPLC, the development of *in vitro* dissolution methods, and PK-PD model development have been described.

## 1. Introduction

Dexibuprofen, S(+)-ibuprofen, is a pharmacologically active form and is more potent than ibuprofen, which has equal quantities of R(−)- and S(+)-enantiomers [1]. Ibuprofen is an NSAID and is widely used to reduce pain, fever, and inflammation. This drug inhibits cyclooxygenases and activates peroxisome proliferators-activated receptors; both of these actions result in reduced inflammation [2–4]. Pharmacokinetic-pharmacodynamic (PK-PD) modeling is a scientific tool to help developers selecte a rational dosage regimen for confirmatory clinical testing. PK/PD modeling can be executed using various approaches, such as direct versus indirect response models and parametric versus nonparametric models. PK/PD concepts can be applied to the individual dose optimization. The limits of PK/PD approaches include the development of appropriate models, the validity of surrogate endpoints, and the acceptance of these models in a regulatory environment. PK-PD modeling allows the estimation of PK-PD parameters and the prediction of these derived, clinically relevant parameters as well. PK-PD simulations allow the assessment of the descriptive parameters as the functions of dose and dose rate. These simulations can provide the dose-response curve for onset, magnitude, and the duration of effect. This information can be valuable in optimizing dose and dosing regimens [5]. Currently, there is a growing recognition of the importance of PK-PD studies in all phases of drug development [6–9]. In preclinical studies, PK-PD is used to interpret toxicokinetics data and via physiological modeling and allometric scaling, it is also used to extrapolate results from animals to humans [10, 11]. During early clinical testing, PK-PD is used to aid in the interpretation of dose-response and escalation studies. In addition, there are several instances in which PK-PD modeling has been used by regulatory agencies to recommend a dose and/or regimen not originally studied as part of the clinical program. As in the case of pharmacokinetics, methods to measure pharmacologic effects and bio-mathematical models had to be developed to characterize and evaluate pharmacodynamic processes.

Mathematical models can be considered as the simplifications of a phenomenon described in terms of an algebraic or differential equation. In the case of PK-PD modeling, it is expected to not only describe, but also predict distinct situations, such as scaling between preclinical to clinical trials, multiple dosing schemes, or different routes of administration [12, 13]. To choose the most appropriate PK-PD model, it is essential to identify the significance of the biological processes involved in eliciting a drug-induced response. Eventually, PK processes, biophase distribution, drug-receptor interaction, signal transduction, and secondary postreceptor events are factors altering the PD behavior of a drug. If that information is available—although only partially—it is possible to link PK and PD with actual physiologic support instead of only abstract numbers. Then, the model-building process involves fitting the available data and the consideration of possible biological differences that usually are translated into inter- and intravariability. In the case of PD variability, it becomes important to identify the useful predictor (covariates) of PD individuality to facilitate the individually optimized pharmacotherapy. It is necessary, therefore, to establish very comprehensive patient profiles during the development of studies. Moreover, the study populations must be representative of the target patient population with respect to age, gender, race, and environmental and pathophysiological characteristics. If these requirements are absent, the relevance and usefulness of covariates may be questionable [14, 15].

Because of the multiple factors intervening in a PK-PD study, it then appears adequate to divide the modeling project into the following two basic blocks such as concerning the clinical or experimental design by itself and the data analysis. Diverse models have been suggested to describe the PK-PD relationship depending upon the nature of drug administration scheme (single doses, multiple doses, long-term infusions, etc.) and the time dependency of PD parameters. Thus, when the system is kinetically at steady state, the concentrations of the active moiety at the active site are constant (after long-term infusions or multiple doses); relatively simple models are needed to characterize the PK-PD relationship. Otherwise, after single doses (nonsteady-state condition) and when time variant PD parameters are present, more complex models are needed to account for phenomena involved in the PK-PD relationship. Approaches such as disequilibrium between biophase and plasma compartment [12], the appearance of active metabolites [16, 17], indirect mechanisms of action [18, 19], sensitization, and tolerance [20–22] have been proposed to explain the apparent dissociation between time courses of concentration and effect. Recently, the combination of powerful nonlinear, mixed effect regression models, statistically robust software tools, and the integration of pharmacokinetic-pharmacodynamic knowledge has permitted optimization of the decision process in therapeutic management. By the incorporation of previous information into these systems, Bayesian forecasting certainly promises the more adequate individualized therapy for a particular patient.

The development and validation of a PK/PD is based on the ability of the fraction of the drug absorbed versus the fraction of the drug-dissolved relationship of various formulations. For the estimation of the drugs present in the biological fluids, HPLC method [23–28] is considered to be more suitable since it is a powerful and rugged method and also extremely specific, linear, precise, accurate, sensitive, and rapid. The present study is developed and validated PK/PD of selected modified release formulations containing dexibuprofen. At present there is no PK/PD studies of the developed formulation have been reported.

## 2. Reagents and Chemicals Used

Acetonitrile, methanol, orthophosphoric acid, sodium acetate, perchloric acid, and triethylamine were supplied by Qualigens Fine Chemicals and S.D. Fine Chemicals. Water (HPLC grade) was obtained from Milli-Q system. All the reagents and chemicals used were of HPLC or analytical grade.

Working standards of dexibuprofen was purchased from Noven Life Sciences (Hyderabad, India) HPMC (Methocel - K100-CR, apparent viscosity, 2% in water at 20°C is 80,000–12000 cP); xanthen and starch 1500 were gift samples from Colorcon Asia Pvt Ltd (Goa, India). Polyvinyl pyrrolidine (PVP-K-30) was a gift sample from Anshul Agencies (Mumbai, India). Aerosil was purchased from Degussa India Pvt Ltd (Mumbai, India).

## 3. Instruments Used

### 3.1. Experimental

This chapter describes the experimental details of the preformulation study, tablet manufacture, bio availability study design and data handling, optimization and validation of the bio analytical methods for the estimation of dexibuprofen in human plasma samples, preparation of standard and sample solutions, development of *in vitro* dissolution methods,* in vitro* data analysis, *in vivo* data analysis, statistical analysis of pharmacokinetic data, and development of pharmacokinetic-pharmacodynamic model.

### 3.2. Preformulation Study

Preformulation in the broadest sense encompasses all the activities and studies that are required to convert an active pharmacological substance into a suitable dosage form. It can be defined as an investigation of the physical and chemical properties of a drug substance alone and also when combined with the excipients. In the present study, therefore, the evaluation of granulations, development of *in vitro* dissolution method, and the compatibility between the drug and the selected polymer were determined.

### 3.3. Development of Dexibuprofen Sustained Release (SR) Tablets

Dexibuprofen SR tablets were prepared by the wet granulation method. All the composition, with the exception of magnesium stearate and aerosol, were thoroughly mixed in a tumbling mixer for 5 min and wetted in a mortar with isopropyl alcohol. The wet mass was sieved (16 mesh) and granules were dried at 40°C for 16 h. The dried granules were sieved (22 mesh) and these granules were lubricated with a mixture of magnesium stearate and aerosil (2 : 1). The dexibuprofen tablets were prepared using an electrically operated punching machine. Compression was performed after granulation process with a single punch press applying a compression force of a 9 KN (preliminary work) or 12 KN (experimental design), equipped with a 12 mm flat-faced punch. For the preliminary work, batches of 100 tablets were prepared. Each batch of experimental design consisted of 100 tablets (drug content in the tablet was 300 mg). Three batches were prepared for each formulation and the compositions of different batches of dexibuprofen SR tablets are given in Table 1. The compressed tablets were evaluated for average weight and weight variation, thickness, diameter, drug content and content uniformity, hardness, friability, disintegration, and *in vitro* drug release.

### 3.4. Pharmacokinetic Studies

Bioavailability studies of the optimized formulations were carried out in crossover design in healthy human volunteers between the developed formulations and the conventional dosage form. The protocol of the study was submitted to the Institutional Human Ethical Committee and the approval for conducting the same was obtained and a prior consent of the volunteers participated in the study was taken. Randomized, two-treatment, two-period, two-sequence, single-dose, crossover bioavailability studies were carried out in healthy human volunteers between the developed sustained release (SR) formulation and the marketed conventional immediate release (IR) formulation to prove the safety and efficacy of the developed SR formulation. A reproducible analytical technique was developed for the estimation of the drugs in the plasma samples. Various pharmacokinetic parameters such as *C*_max⁡_, *T*_max⁡_, *t*_1/2_, *k*_el_, AUC_0–*t*_, and AUC_0–∞_ were estimated.

### 3.5. Pharmacodynamic Model for Dexibuprofen

#### 3.5.1. Visual Analogue Scale

A visual analogue scale (VAS) is a measurement instrument that tries to measure a characteristic or attitude that is believed to range across a continuum of values and cannot easily be directly measured. For example, the amount of pain that a patient feels ranges across a continuum from none to an extreme amount of pain. From the patient's perspective, this spectrum appears continuous ±; their pain does not take discrete jumps, as a categorization of none, mild, moderate, and severe would suggest. It was to capture this idea of an underlying continuum that the VAS was devised. Operationally a VAS is usually a horizontal line, 100 mm in length, anchored by word descriptors at each end (see Figure 6). 

The patients mark on the line the point that they feel it represents their perception of their current state. The VAS score is determined by measuring in millimetres from the left hand end of the line to the point that the patient marks. There are many other ways in which VAS has been presented, including vertical lines and lines with extra descriptors. Wewers and Lowe [29] provide an informative discussion of the benefits and shortcomings of different styles of VAS. As such an assessment is clearly highly subjective, these scales are of most value when looking at change within individuals and are of less value for comparing across a group of individuals at one time point. It could be argued that a VAS is trying to produce interval/ratio data out of subjective values that are at best ordinal. Thus, some caution is required in handling such data. Many researchers prefer to use a method of analysis that is based on the rank ordering of scores rather than their exact values, to avoid reading too much into the precise VAS score.

## 4. Results and Discussion

This section describes the experimental results obtained in the present investigation in the form of tables and figures along with a detailed analysis on the results of preformulation study, tablet manufacture, bioavailability study design, data handling, optimization and validation of the bio analytical methods for theestimationofdexibuprofen in human plasma samples, amount of the selected drugs present in plasma samples, *in vitro* dissolution method, determination of pharmacokinetic parameters, statistical evaluation, *in vivo* and *in vitro* data analysis, and pharmacodynamic model.

### 4.1. Development of Dexibuprofen SR Tablets

**Figure 1:**
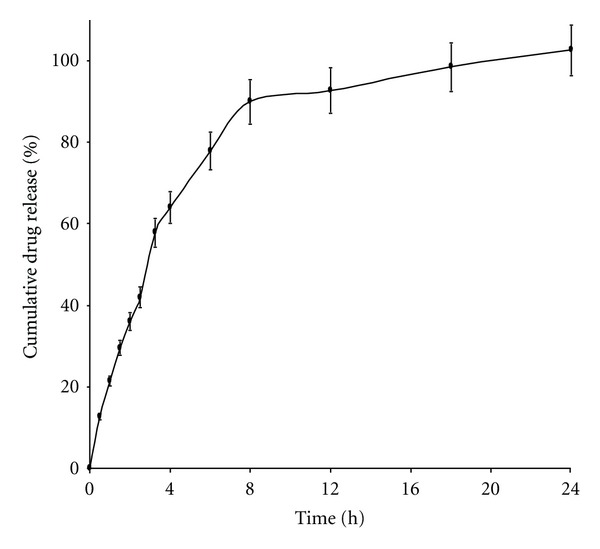
Release profiles of dexibuprofen from xanthen (polymer) containing formulation (F_10_).

The physical properties of different batches of developed tablets are given in Table 1 and Figure 1 of dexibuprofen, respectively. All the batches showed a uniform thickness. The average percentage deviation of 20 tablets of each formula was less than ±5% and hence all formulations passed the test for uniformity of weight as per official requirements (Pharmacopoeia of India 1996). Good uniformity content was found among three different batches of tablets. Another measure of tablets strength is friability. In the present study, the percentage friability for all the formulations was below 1%, indicating that the friability is within the prescribed limits. All the tablets formulations showed acceptable pharmaco technical properties and complied with the specifications for weight variation, drug content, hardness, and friability.

A single-dose, randomized, complete, two-treatment crossover study was conducted in healthy human subjects for the selected drug formulations. Six volunteers aged between 20 and 30 years were selected. Seven days prior to the commencement of the study, volunteers were subjected to preliminary screening and standard clinical and biochemical investigations.

### 4.2. Bioavailability Studies

**Figure 2:**
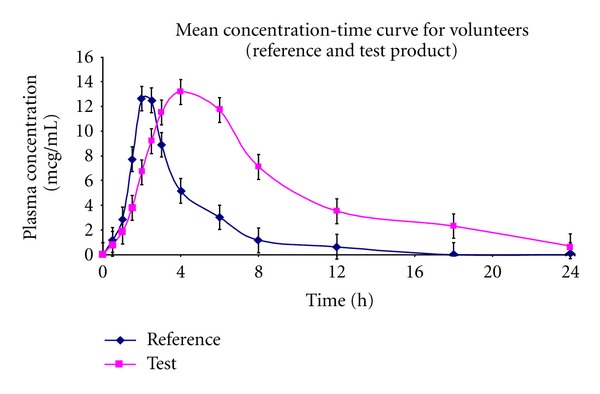
Mean plasma concentration-time profile of dexibuprofen from developed sustained release tablets (test) and marketed immediate release tablet (reference).

After overnight fasting, the volunteers were given code numbers and allocated to the treatment in accordance with the randomized code. The order of treatment administration was randomized in two sequences (AB and BA) in blocks of two. In each dosing session, volunteers received reference product A (immediate release formulations) and Test B (sustained release formulations). A wash-out period of seven days was allowed between dose administrations. Blood samples (4 mL) were collected at 0 (before drug administration), 0.5, 1.0, 1.5, 2.0, 2.5, 3.0, 4.0, 6.0, 8.0, 12.0, 18.0, and 24.0 h after dosing. The samples were centrifuged and plasma was separated. There were no serious adverse effects observed during the entire study (Table 2 and Figure 2).

### 4.3. Pharmacokinetic Data Analysis

Pharmacokinetic parameters such as peak plasma concentration (*C*_max⁡_), time to peak concentration (*t*_max⁡_), area under the plasma concentration-time curve (AUC_0–*t*_ and AUC_0–∞_), elimination rate constant (*k*_el_), and elimination half-life (*t*_1/2_) were calculated separately and the blood level data of selected formulations were compared.

### 4.4. Statistical Analysis

The pharmacokinetic parameters of two different drug formulations of dexibuprofen was compared statistically by one-way ANOVA (analysis of variance) using SPSS version 16.0. *P* value of <0.05 was considered as statistically significant. The results were expressed as the mean ± SD. The pharmacokinetic parameters *C*_max⁡_, AUC_0–*t*_, and AUC_0–∞_ of the immediate release and sustained release formulations of dexibuprofen were found to be significantly different by one-way ANOVA.

### 4.5. Pharmacodynamic Study of Dexibuprofen

**Figure 3:**
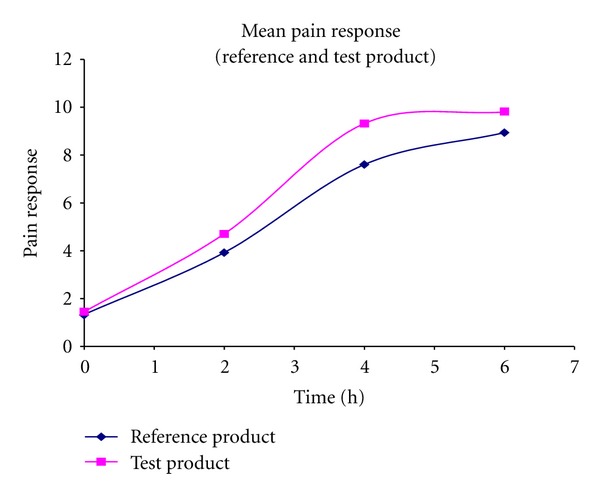
Mean pain response (reference and test product).

**Figure 4:**
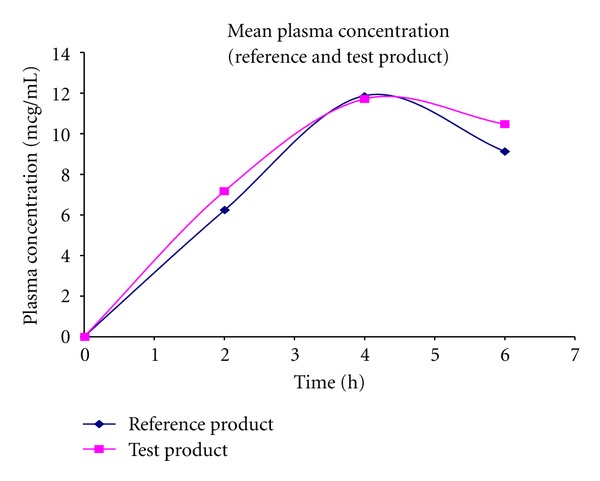
Mean plasma concentration (reference and test product).

**Figure 5:**
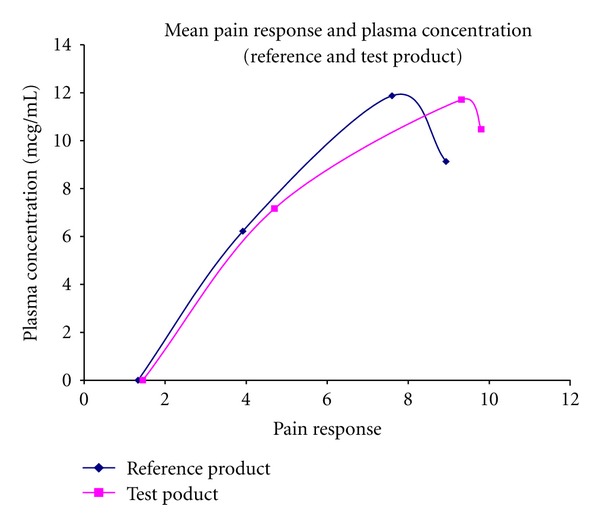
Mean pain response and plasma concentration (reference and test product).

**Figure 6:**
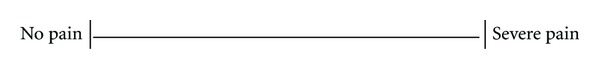


The results indicated by the pain scale [29] of the developed dexibuprofen SR tablets have shown notable pain relief when compared to the marketed IR tablets. The quantification of the chromatogram was performed using peak area ratios (response factor) of the drug to an internal standard. The individual and mean concentration of the drugs present in the plasma samples were calculated and are presented in Tables 3 and 4 and illustrated in Figures 3, 4, and 5.

## 5. Conclusion

Based on these observations, it is concluded that the formulated matrix tablets containing dexibuprofen are capable of exhibiting sustained release properties, stable and feasible for industrial scale production. Thus they are capable of reducing the dose intake, minimize the blood level oscillations, dose-related adverse effects and cost, and ultimately improve the patient compliance in the therapeutic management of pain and hypertension. It is also concluded that the present PK/PD studies have demonstrated that pain and blood pressure management were found to be effective in developed SR formulations of dexibuprofen as compared with marketed immediate release formulations. Further studies involving their suitability for long-time application, shelf life determination, bioavailability, and clinical investigations in large populations may, however, be necessary to further establish its potential and therapeutic efficacy.

## Figures and Tables

**Table 1:** Formulation prepared by wet granulation method (F_1_–F_10_) for dexibuprofen.

F^a^	DXI	HPMC	Xanthen	Avicel PH 102	Magnesium stearate	Aerosil	PVP-k-29/32	Total (mg/tab)
F_1_	300	37.5	—	130	5	2.5	25	500
F_2_	300	75	—	92.5	5	2.5	25	500
F_3_	300	112.5	—	55	5	2.5	25	500
F_4_	300	150	—	17.5	5	2.5	25	500
F_5_	300	—	112.5	55	5	2.5	25	500
F_6_	300	—	37.5	130	5	2.5	25	500
F_7_	300	—	75	92.5	5	2.5	25	500
F_8_	300	—	150	17.5	5	2.5	25	500
F_9_	300	—	50	117.5	5	2.5	25	500
F_10_	300	—	12.5	160	5	2.5	25	500

^a^Code of formulations.

**Table 2:** Mean pharmacokinetic profile (*n* = 6).

Drug name	*C*_max⁡_	*T*_max⁡_	AUC_0–*t *_	*t*_1/2_	*k*_el_	AUC_0–∞_
IR	13.812 (1.072)	2.25 (0.273)	45.591 (6.331)	2.188 (0.175)	0.318 (0.024)	47.621 (6.242)

SR	14.178 (0.701)	5.00 (1.095)	117.843 (14.537)	4.772 (0.303)	0.145 (0.008)	122.620 (14.552)

^a^Immediate release (IR) tablets.

^b^Sustained release (SR) tablets.

^c^Results represent the mean of replicate determination with the standard deviation given in parenthesis.

^†^Significantly higher than IR tablets.

^‡^Significantly lower than IR tablets.

**Table 3:** Pain response versus plasma concentration of dexibuprofen (test product).

Time points (h)	Pain response						Plasma concentration (*μ*g/mL)					
	V_1_^a^	V_2_^a^	V_3_^a^	V_4_^a^	V_5_^a^	V_6_^a^	V_1_^a^	V_2_^a^	V_3_^a^	V_4_^a^	V_5_^a^	V_6_^a^
0	1.1	0.5	2.3	1.4	2.4	1	0	0	0	0	0	0
2	5.1	3.5	5.4	4.4	5.7	4.1	7.1254	7.5624	5.2039	8.1283	6.9584	7.9892
4	9.3	8.6	9.4	9.1	9.7	9.8	13.5241	10.0548	12.5264	11.9856	9.5321	12.6354
6	9.9	9.6		9.8	9.9		9.1032	12.1627	9.9287	9.9012	12.9658	8.7541

^a^Volunteers code.

**Table 4:** Pain response versus plasma concentration of dexibuprofen (reference product).

Time points (h)	Pain response						Plasma concentration (mcg/mL)					
	V_1_^a^	V_2_^a^	V_3_^a^	V_4_^a^	V_5_^a^	V_6_^a^	V_1_^a^	V_2_^a^	V_3_^a^	V_4_^a^	V_5_^a^	V_6_^a^
0	0.7	0.6	1.7	1.6	2.1	1.3	0	0	0	0	0	0
2	4.3	2.9	4.3	3.7	4.6	3.7	5.9826	6.3614	4.6985	7.4978	7.3956	5.3869
4	8.9	7.5	6.9	7.4	8.5	6.4	12.4958	11.3057	12.0498	10.2584	12.0394	13.0695
6	9.2	8.5	8.7	9	9.3	8.9	8.9375	9.5473	8.9573	8.5893	9.2738	9.4752

^a^Volunteers code.
